# NLRP3 Inhibition Reduces rt-PA Induced Endothelial Dysfunction under Ischemic Conditions

**DOI:** 10.3390/biomedicines10040762

**Published:** 2022-03-24

**Authors:** Maximilian Bellut, Anthony T. Raimondi, Axel Haarmann, Lena Zimmermann, Guido Stoll, Michael K. Schuhmann

**Affiliations:** Department of Neurology, University Hospital of Wuerzburg, Josef-Schneider-Str. 11, 97080 Würzburg, Germany; bellut_m@ukw.de (M.B.); anthony.t.raimondi@gmail.com (A.T.R.); haarmann_a@ukw.de (A.H.); papp_l@ukw.de (L.Z.); stoll_g@ukw.de (G.S.)

**Keywords:** NLRP3, inflammasome, MCC950, rt-PA, blood–brain barrier, Cell Index, ASC, ischemic stroke, i.v. thrombolysis

## Abstract

Thrombolysis with recombinant tissue plasminogen activator (rt-PA) is a mainstay of acute ischemic stroke treatment but is associated with bleeding complications, especially after prolonged large vessel occlusion. Recently, inhibition of the NLRP3 inflammasome led to preserved blood–brain barrier (BBB) integrity in experimental stroke in vivo. To further address the potential of NLRP3 inflammasome inhibition as adjunct stroke treatment we used immortalized brain derived endothelial cells (bEnd5) as an in vitro model of the BBB. We treated bEnd5 with rt-PA in combination with the NLRP3 specific inhibitor MCC950 or vehicle under normoxic as well as ischemic (OGD) conditions. We found that rt-PA exerted a cytotoxic effect on bEnd5 cells under OGD confirming that rt-PA is harmful to the BBB. This detrimental effect could be significantly reduced by MCC950 treatment. Moreover, under ischemic conditions, the Cell Index—a sensible indicator for a patent BBB—and the protein expression of Zonula occludens 1 stabilized after MCC950 treatment. At the same time, the extent of endothelial cell death and NLRP3 expression decreased. In conclusion, NLRP3 inhibition can protect the BBB from rt-PA-induced damage and thereby potentially increase the narrow time window for safe thrombolysis in stroke.

## 1. Introduction

Ischemic stroke (IS) is one of the leading causes of death and disability worldwide [1]. Thrombolysis and mechanical thrombectomy are applied early after stroke onset to achieve rapid recanalization as a prerequisite for a good functional outcome. However, rapid infarct growth from stroke onset until the initiation of these interventions increases the probability of futile or even harmful recanalization, mainly due to bleeding complications [2]. In particular, the application of recombinant tissue plasminogen activator (rt-PA) increases the risk of hemorrhagic stroke transformation beyond 4.5 h of stroke onset. Furthermore, there is experimental evidence that rt-PA can impair the blood–brain barrier (BBB) [3,4,5,6].

Inflammasomes are molecular protein complexes which sense cellular deviation from homeostasis and subsequently initiate inflammatory responses [7]. Inflammasomes consist of three components: firstly, a cytosolic pattern recognition receptor (PRR)—most notably the NOD-, LRR-, and pyrin domain-containing protein 3 (NLRP3)—causing the oligomerization of the sensor by recognizing inflammatory mediators and DAMPs in sterile inflammatory processes such as IS [8,9]. Secondly, the recruitment of ASC (apoptosis-associated speck-like protein containing a caspase recruitment domain) is induced. Thirdly, ASC filaments provide a docking site for pro-caspase 1 as effector molecule [10,11]. This triggers the autoactivation of caspase 1 and the downstream activation of the pro-inflammatory cytokines IL-1β and IL-18 as well as the pyroptosis-inducing protein Gasdermin D [12,13], which are significantly expressed during IS [14,15,16].

We could recently show that NLRP3 inflammasome is upregulated early after stroke onset in neurons and glial cells, but also in endothelial cells (EC) [16]. This upregulation is of particular interest, as EC die under hypoxic conditions. This leads to a BBB breakdown, which responds to NLRP3 inhibition [15]. Our goal, now, was the quantification of the endothelial barrier function in the bEnd5 cell model dependent upon the period of oxygen and glucose deprivation (OGD) and additional rt-PA administration. Furthermore, we tested whether NLRP3 inhibition can mitigate rt-PA induced damage of the BBB.

## 2. Materials and Methods

### 2.1. Materials

A total of 10 mg of the inflammasome-inhibitor MCC950 (NLRP3 Inhibitor, MCC950, #538120, Merck, Darmstadt, Germany) were dissolved in 1 mL 1× phosphate-buffered saline (PBS) (Dulbecco′s Phosphate Buffered Saline, #D8537, Merck). MCC950 was further diluted with cell culture medium to 100 µmol/L. rt-PA (Actilyse, PZN-03300636, Boehringer Ingelheim, Ingelheim am Rhein, Germany) was diluted with cell culture medium to a final concentration of 50 ng/mL in line with the average concentration of rt-PA detected in human pial blood samples [17]. Dulbecco’s Modified Eagle’s Medium (DMEM) high glucose (4.5 g/L) (Dulbecco′s Modified Eagle′s Medium—high glucose, #D5671), DMEM low glucose (1 g/L) (Dulbecco′s Modified Eagle′s Medium—low glucose, #D5921), sterile water (Water, #W3500), L-Glutamine (L-Glutamine Solution 200 mM, #59202C), Trypsin (Trypsin-EDTA solution 0.25%, #T4049), and PI (Propidium iodide solution—solution 1.0 mg/mL in water, #P4864) were all purchased by Merck. Calf serum (FCS) (Sterile Plasma Derived Bovine Serum, #60-00-850) was provided by First Link (UK) Ltd. (Birmingham, UK). The T75-cell culture flasks (Cellstar Cell Culture Flasks, 75 cm^2^, #658170) were provided by Greiner Bio-One GmbH (Frickenhausen, Germany), the 96-well plates (Nunc™ Cell-Culture, 96-Well, #165306), DAPI (ProLong™ Gold Antifade Mountant with DAPI, #P36931) and as secondary antibodies Alexa Fluor^TM^ 488 goat anti-mouse IgG (Goat anti-Mouse IgG (H+L), Alexa Fluor 488, #A11001) and Alexa Fluor^TM^ 546 goat anti-rabbit IgG (Goat anti-Rabbit IgG (H+L), Alexa Fluor 546, #A11035) were provided by Thermo Fisher Scientific (Waltham, MA, USA). For the xCELLigence experiments, xCELLigence E-Plates (xCELLignece E-Plate VIEW 16, #300601140) were purchased by Agilent (Santa Clara, CA, USA).

### 2.2. Cell Culture

We purchased bEnd5 cells from Merck (bEnd5, #96091930). They were grown in DMEM (high glucose, 4.5 g/L), supplemented with 10% FCS and 1% L-Glutamine (200 mM), in a humidified (95%) 37 °C incubator with 5% CO_2_ and 21% O_2_. We plated bEnd5 in 75 cm^2^ culture flasks and subcultured them using 0.25% (*w*/*v*) trypsin in 0.02% (*w*/*v*) EDTA at 80% confluence. We changed media every 2 d. We passaged the cells chosen for experimentation between 18 and 26 times. For resistance measurements, we transferred bEnd5 cells on gold electrode plates (ACEA, San Diego, CA, USA) and for immunofluorescent microscopy on 96-well plates 24 h prior to commencement of the respective trials.

### 2.3. Oxygen and Glucose Deprivation (OGD)

We exposed confluent monolayers of bEnd5 cells to hypoxic (1% O_2_, 95% humidity, 5% CO_2_, 37 °C) and aglycemic conditions by replacing the culture medium with low glucose-medium (hypoxic DMEM low glucose with 1% L-Glutamine without FCS). We preincubated the OGD medium for 24 h under hypoxic conditions before administration.

### 2.4. Treatment Regimes

We used a rt-PA concentration of 50 ng/mL corresponding to the average value of recently analyzed human post occlusive blood samples [17].

A total of 100 µmol/L of the specific NLRP3 inflammasome-inhibitor MCC950 were used to treat bEnd5 cells. MCC950 was dissolved as described above. As vehicle treatment, the same amount of pure culture or OGD medium was added. The investigators were blinded to the group allocation.

### 2.5. xCELLigence Assay

For label-free real-time assessment of transendothelial resistance, we used an Agilent xCELLigence RTCA DP system. It records the impedance changes compared to the background of cell-free electrodes at three different alternating current frequencies expressed as the dimensionless Cell Index [18]. It correlates to the transendothelial electrical resistance but does additionally reflect the capacitance of the cell layer. When confluent after 24 h of growth, evidenced by a plateau of the Cell Index, we treated the cells with MCC950, rt-PA, MCC950 and rt-PA at the same time, or rather vehicle, under either normoxic or OGD conditions as indicated. Data was processed by the xCELLigence RTCA DP software 1.2.1 (Agilent, Santa Clara, CA, USA).

### 2.6. Immunofluorescence Microscopy

For histology we fixated bEnd5 cultures with a glyoxal solution containing 40% glyoxal, acetic acid, water, and 100% ethanol. Cell cultures were dyed with DAPI, PI, and antibodies against NLRP3 (anti-NLRP3/NALP3, mAb (Cryo-2), #AG-20B-0014, 1:100, Adipogen Life Sciences, San Diego, CA, USA), Zonula occludens 1 (ZO-1 Polyclonal Antibody, #61-7300, 1:1000, Thermo Fisher Scientific), and ASC (Anti-ASC (AL177), #AG-25B-0006, 1:100, Adipogen). We used secondary antibodies in a dilution of 1:100. For recording, we used a Leica microscope (Leica DMi8, DMC 2900/DFC 3000G camera control, LAS X software (Leica, Wetzlar, Germany)). To measure cell death bEnd5 cells cultivated on 96-well plates were visualized with transmitted light microscopy and apoptotic cells with fluorescence measurements after PI (1:200) staining with the above-mentioned microscope. The red fluorescent cells were counted. For measurement of NLRP3, ASC, and ZO-1 intensity, images of the cell cultures were recorded with the same microscope. Subsequently, after converting the images into 16-bit black and white files, the intensities of the respective stainings were determined with ImageJ Analysis Software 1.52a (National Institutes of Health, Bethesda, MD, USA).

### 2.7. Statistics

Results are presented as grouped summary data indicating median and standard deviation for each time point. For statistical analysis the GraphPad Prism 8 software (GraphPad Company, San Diego, CA, USA) was used. Data was tested for Gaussian distribution with the D’Agostino-Pearson omnibus normality test and then analyzed by 1-way analysis of variance (ANOVA) with post hoc Tukey adjustment for *p* values. Probability values < 0.05 were considered to indicate statistically significant results.

## 3. Results

### NLRP3 Inhibition Improves Endothelial Barrier Function and EC Survival after rt-PA Administration under OGD

**Figure 1:**
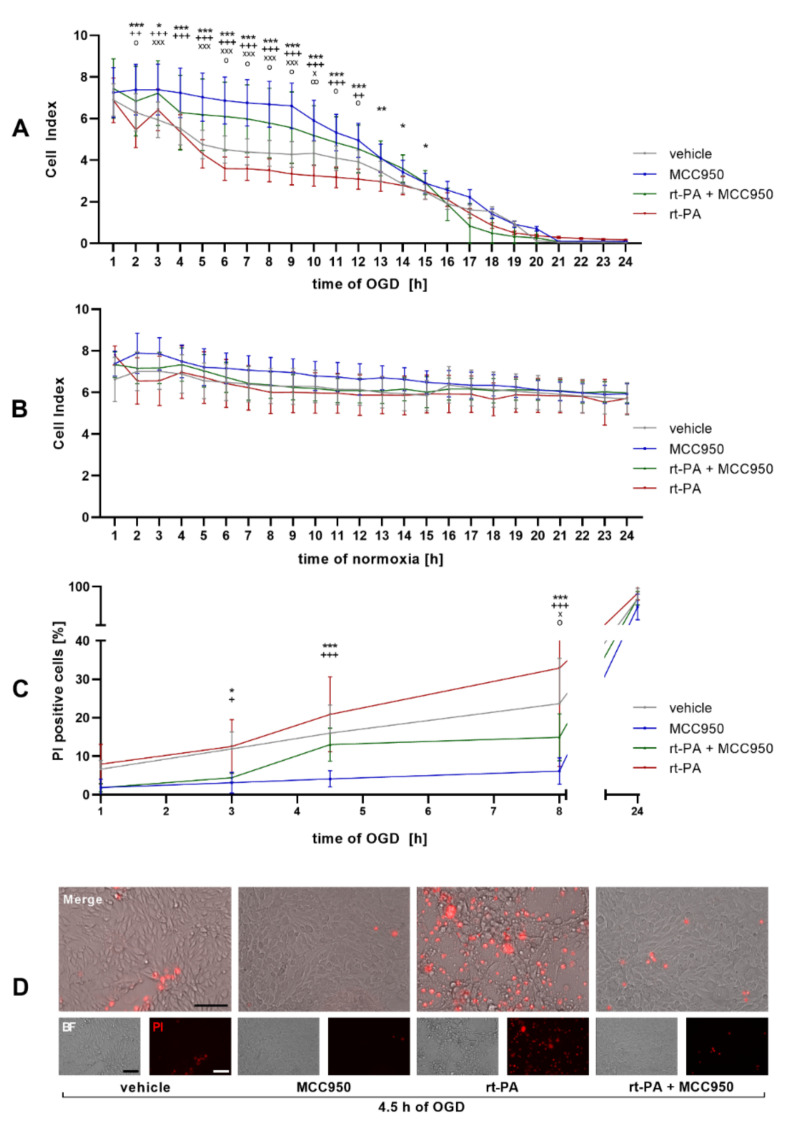
NLRP3 inhibition stabilizes the Cell Index of endothelial cells and reduces endothelial cell death under OGD. (**A**) The Cell Index over a period of 24 h of OGD (1% O_2_, 5% CO_2_, 95% humidity, 37 °C, 1 g/L glucose) as measured with the ACEA xCELLigence DP system. bEnd5 were either treated with vehicle, MCC950, rt-PA, or rt-PA and MCC950 (*n* = 24 out of 3 independent experiments). (**B**) The Cell Index over a period of 24 h of normoxia. bEnd5 were either treated with vehicle, MCC950, rt-PA, or rt-PA and MCC950 (*n* = 24 out of 3 independent experiments). (**C**) Percentage of apoptotic, propidium iodide (PI) positive, bEnd5 cells per well after 1 h, 3 h, 4.5 h, 8 h, and 24 h of OGD depending on the treatment regime (*n* = 15 out of 3 independent experiments). (**D**) Representative microscopic brightfield images of bEnd5 after 4.5 h of OGD and visualization of treatment-dependent PI (red) uptake; 10× objective; scale bar = 100 µm. Data was analyzed by 1-way ANOVA with post hoc Tukey adjustment * *p* < 0.05; ** *p* < 0.01; and *** *p* < 0.001. Significance as indicated by * refers to the comparison between the rt-PA treated group and the rt-PA + MCC950 treated group, by ^+^, ^++^ *p* < 0.01, ^+++^ *p* < 0.001 to the comparison between the vehicle treated group and the MCC950 treated group, by ^ο^, ^οο^ *p* < 0.01 to the comparison between the vehicle treated group and the rt-PA treated group and by ^x^, ^xxx^ *p* < 0.001 to the comparison between vehicle and the rt-PA + MCC950 treated group. Merged images are enlarged by a factor of 2.

**Figure 2:**
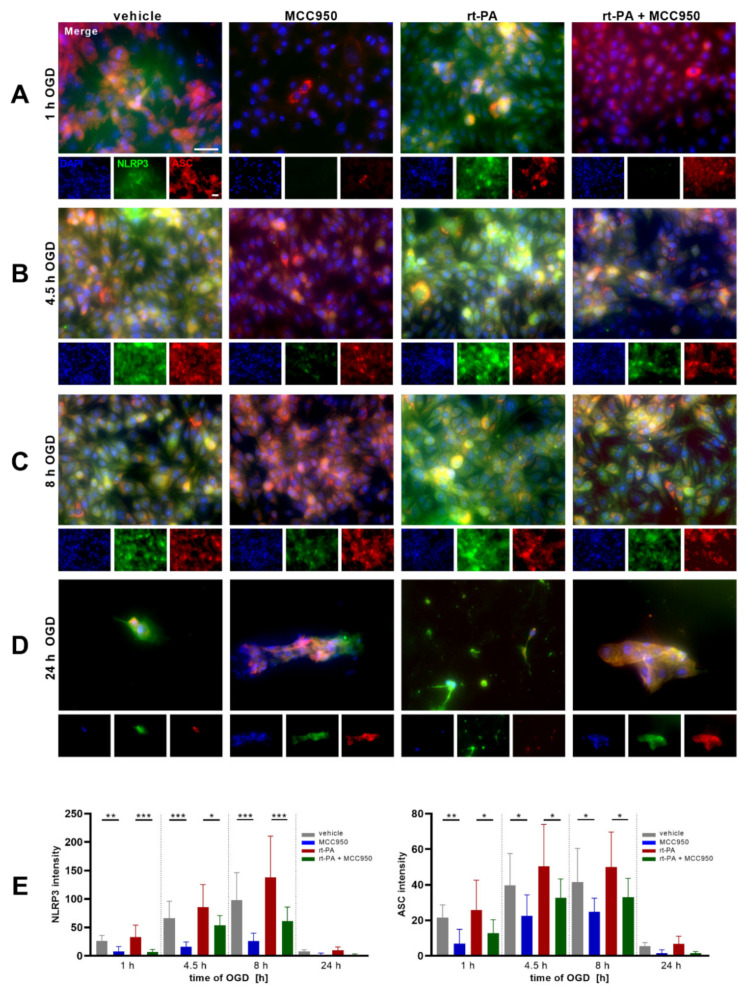
MCC950 treatment reduces NLRP3 and ASC expression under OGD. Representative NLRP3 (green), ASC (red), and DAPI (blue) immunofluorescence stainings of bEnd5 after (**A**) 1 h, (**B**) 4.5 h, (**C**) 8 h, or (**D**) 24 h of OGD (1% O_2_, 5% CO_2_, 95% humidity, 37 °C, 1 g/L glucose) either with vehicle, MCC950, rt-PA, or dual rt-PA and MCC950 treatment. (**E**) Quantification of NLRP3 (left) and ASC (right) expression by intensity measurement of the immunofluorescent stainings (*n* = 15 out of 3 independent experiments); 20 × objective; scale bar = 20 µm; scale bars account for all images. Merged images are enlarged by a factor of 3. Data was analyzed by 1-way ANOVA with post hoc Tukey adjustment. * *p* < 0.05; ** *p* < 0.01; and *** *p* < 0.001.

The Cell Index is a sensitive read-out for endothelial monolayer integrity. Using the xCELLigence real-time cell analysis (RTCA) system an accelerating decrease in the Cell Index could be detected under OGD, which amplified significantly after rt-PA administration. Coincubation with MCC950, though, stabilizes the Cell Index of the rt-PA + MCC950 group until 15 h of OGD in comparison to rt-PA administration only (Figure 1A). Under normoxic conditions no significant differences between the single treatment regimens stood out (Figure 1B). In a next step, the Cell Index under OGD was correlated with the quantified cell death: while the number of dead EC was the highest in the rt-PA treatment group with a significant reduction in the rt-PA + MCC950 group, the cell death was lowest in the MCC950 only group, as shown by propidium iodide (PI) staining (Figure 1C,D). To verify the role of NLRP3 within the loss of endothelial barrier function during OGD and rt-PA treatment, an immunofluorescent microscopic analysis of the cell culture was performed. It showed an increasing NLRP3 signal during OGD incubation in the vehicle group with a considerable gain of NLRP3 intensity in the rt-PA group and an unambiguous reduction in the respective MCC950 groups. After 1 h of OGD only a faint NLRP3 signal was detected in the MCC950 only group. The staining of the apoptosis-associated speck-like protein containing a CARD domain (ASC), the essential adaptor molecule for inflammasome activation, which is described as a readout parameter for inflammasome activation, showed similar results (Figure 2) [19].

**Figure 3:**
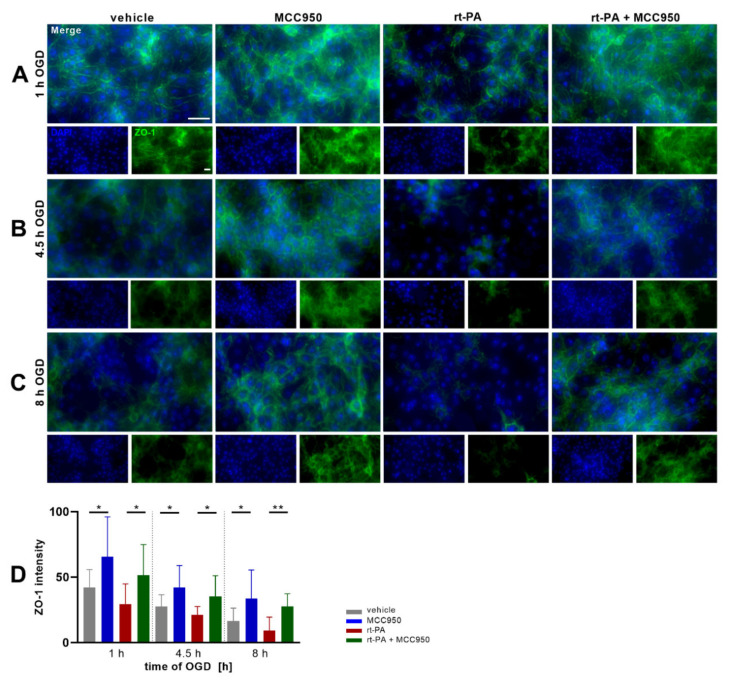
MCC950 treatment improves ZO-1 expression under OGD. Representative ZO-1 (green) and DAPI (blue) immunofluorescence stainings of bEnd5 after (**A**) 1 h, (**B**) 4.5 h, or (**C**) 8 h of OGD (1% O_2_, 5% CO_2_, 95% humidity, 37 °C, 1 g/L glucose) either with vehicle, MCC950, rt-PA or dual rt-PA and MCC950 treatment. (**D**) Quantification of ZO-1 expression by intensity measurement of the immunofluorescent stainings (*n* = 15 out of 3 independent experiments); 20× objective; scale bar = 20 µm; scale bars account for all images. Merged images are enlarged by a factor of 2. Data was analyzed by 1-way ANOVA with post hoc Tukey adjustment. * *p* < 0.05; ** *p* < 0.01.

The characteristic high transendothelial resistance of brain endothelial monolayers is achieved by the formation of tight junctions (TJ) that seal the intercellular clefts. These transmembraneous multi-protein complexes are linked to the cytoskeleton by intracellular adapter proteins such as zonula occludens-1 (ZO-1). Thus, ZO-1 reduction after OGD exposure and rt-PA stimulation, as analyzed by immunofluorescent microscopic analysis, points to a loss of TJ integrity. After 8 h of OGD, the vehicle group showed a distinctly reduced ZO-1 signal while it vanished nearly completely in the rt-PA group. In the MCC950 treated groups, though, a distinct intensity remained even after 8 h of OGD (Figure 3).

## 4. Discussion

Intravenous rt-PA treatment in acute ischemic stroke is restricted to 4.5 h after stroke onset, partly due to an increasing risk of side effects, such as bleeding complications, with progressing occlusion time. As our principal finding, we show that targeting the NLRP3 inflammasome can protect brain endothelial cells from unwanted toxic side effects of the thrombolytic agent rt-PA under ischemic conditions.

Prolonged OGD of bEnd5 cells, as a model system of the brain microvasculature during large vascular occlusion in IS, triggers cell death over time [15]. This is accompanied by a rise in NLRP3, as well as ASC protein expression and the disruption of endothelial barrier function. Of note, further deterioration of hypoxic endothelial damage by rt-PA treatment was significantly attenuated when MCC950 was co-administered. Importantly, to achieve the greatest possible transferability of the in vitro model, the very same concentration of rt-PA was applied to the cells that was averagely measured in IS patients behind the occluding thrombus before mechanical thrombectomy [17]. The observed disruption of barrier integrity in the rt-PA treated group within 6 h of OGD resembles data of human rt-PA observational studies. Here, the number needed to treat (NNT) rises from 10 (3 h) to 19 (4.5 h) and further to 50 (6 h), while the number needed to harm equals the NNT after 6 h [20].

In the given in vitro setting, clinically important variables such as symptom severity, possible side effects of the treatment, or the overall outcome cannot be examined, which we consider as a study limitation [21]. Furthermore, to evaluate detailed molecular mechanisms of NLRP3 activation and corresponding downstream inflammatory processes when endothelial cells are exposed to rt-PA under ischemic conditions, additional studies are needed.

Taken together, our study provides evidence that early application of a NLRP3 inhibitor does not only preserve the patency of the BBB in middle cerebral artery occlusion, but furthermore protects hypoxic endothelial cells from side effects of concomitant rt-PA treatment. Therefore, NLRP3 inhibition presents as a promising therapeutic target to extend the narrow window of opportunity for acute stroke treatment.

## Data Availability

Any additional data will be provided upon reasonable request.
